# Vitamin D Binding Protein: A Historic Overview

**DOI:** 10.3389/fendo.2019.00910

**Published:** 2020-01-10

**Authors:** Roger Bouillon, Frans Schuit, Leen Antonio, Fraydoon Rastinejad

**Affiliations:** ^1^Laboratory of Clinical and Experimental Endocrinology, Department of Chronic Diseases, Metabolism and Ageing, KU Leuven, Leuven, Belgium; ^2^Gene Expression Unit, Department of Cellular and Molecular Medicine, KU Leuven, Leuven, Belgium; ^3^Department of Endocrinology, University Hospitals Leuven, Leuven, Belgium; ^4^Nuffield Department of Medicine, Target Discovery Institute, University of Oxford, Oxford, United Kingdom

**Keywords:** vitamin D, vitamin D binding protein (DBP), megalin, actin, 25-hydoxyvitamin D, 1,25-dihydoxyvitamin D

## Abstract

Vitamin D and all its metabolites are bound to a specific vitamin D binding protein, DBP. This protein was originally first discovered by its worldwide polymorphism and called Group-specific Component (GC). We now know that DBP and GC are the same protein and appeared early in the evolution of vertebrates. DBP is genetically the oldest member of the albuminoid family (including albumin, α-fetoprotein and afamin, all involved in transport of fatty acids or hormones). DBP has a single binding site for all vitamin D metabolites and has a high affinity for 25OHD and 1,25(OH)2D, thereby creating a large pool of circulating 25OHD, which prevents rapid vitamin D deficiency. DBP of higher vertebrates (not amphibians or reptiles) binds with very high affinity actin, thereby preventing the formation of polymeric actin fibrils in the circulation after tissue damage. Megalin is a cargo receptor and is together with cubilin needed to reabsorb DBP or the DBP-25OHD complex, thereby preventing the urinary loss of these proteins and 25OHD. The total concentrations of 25OHD and 1,25(OH)2D in DBP null mice or humans are extremely low but calcium and bone homeostasis remain normal. This is the strongest argument for claiming that the “free hormone hypothesis” also applies to the vitamin D hormone, 1,25(OH)2D. DBP also transports fatty acids, and can play a role in the immune system. DBP is genetically very polymorphic with three frequent alleles (DBP/GC 1f, 1s, and 2) but in total more than 120 different variants but its health consequences, if any, are not understood. A standardization of DBP assays is essential to further explore the role of DBP in physiology and diseases.

## Introduction

The vitamin D binding protein (DBP) is a multifunctional protein that is well-conserved in the evolution of vertebrates. We review the history of the discovery of this gene and protein and summarize its main functions as known today. The major milestones in the discovery of this gene and protein and in the elucidation of its function are summarized in Table 1.

**Table 1:** Major milestones in the discovery of the role of the vitamin D binding protein.

1	Antirachitic activity in serum migrates with α-globulin mobility	(1)
2	Radioactive vitamin D or 25OHD migrate with different electrophoretic mobility in serum from mammals, birds, amphibian or fish (α or β-globulin or albumin mobility)	(2)
3	Discovery of a major serum protein with genetically different polymorphism called, Group-specific Component (GC)	(3)
4	Identity of GC with the vitamin D binding protein of human serum	(4)
5	Purification and characterization of human DBP and other species, including high affinity-high capacity properties van DBP from higher vertebrate species	(5–7)
6	Immunoassays of DBP in humans and other species	(8)
7	DBP is a member of the albuminoid family	(9)
8	Description of the gene (and protein) structure of GC/DBP	(10)
9	DBP binds actin and plays a crucial role in depolymerization of extracellular actin filaments	(11)
10	Sex hormones regulate the concentration of DBP in a species-specific way. The concentration of total 1,25(OH)_2_D fluctuates in line with the total concentration of DBP so that free 1,25(OH)_2_D is feedback regulated	(12, 13)
11	Free 25OHD concentrations are extremely low (<0.1 % of total concentration) and not feed-back regulated	(14)
12	DBP binds to megalin at the luminal site of renal tubuli and thereby avoid urinary loss of 25OHD	(15)
13	DBP null mice are viable and healthy but have extremely low total concentrations of 25OHD and 1,25(OH)_2_D	(16)
14	DBP binds fatty acids, and unsaturated fatty acids impair the binding of 1,25(OH)_2_D and 25OHD to DBP	(17, 18)
15	DBP binds to membranes proteoglycans of leucocytes and thereby enhances complement C5a-stimulated chemotactic activity in activated neutrophils	(19)
16	Crystal structure of human DBP	(20)
17	DBP can be measured by mass-mass spectrometry	(21, 22)
18	First human subject with homozygous deletion of the GC gene	(23)

## Discovery of GC Protein as an α-globulin and Its Identity With the Serum Vitamin D Binding Protein

**Figure 1:**
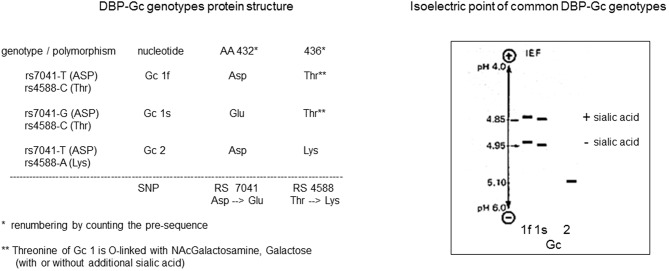
DBP/GC polymorphism as revealed by measurement of their isoelectric point and protein structure.

In 1961, when serum proteins were still mainly characterized by their electrophoretic mobility as α, β, or γ globulins, further identification of the major proteins led to the discovery a highly polymorphic protein with genetically defined small differences in electrophoretic mobility, and therefore named “Group-specific component” or GC (24). Initially, only GC1 and GC2 were identified, but later on GC1 was found to be a mixture of GC1f (fast) and GC1s (slow), because GC1f has a slightly faster electrophoretic mobility than GC1s (Figure 1). Using polyclonal antibodies to detect GC and due to improved sensitivity for detecting small differences in the isoelectric point by isoelectric focusing of sera from subjects from around the world, more than 120 different variants were detected (Figure 1) (25–27). This technique allowed using this protein to study genetic links between populations and to use it in forensic medicine or paternity disputes (28).

Many authors studied independently the electric mobility of “vitamin D” activity in serum either by measuring the anti-rachitic activity, or later on, by using radiolabeled vitamin D or 25-hydroxyvitamin D (25OHD). Based on such data, lipoproteins were found to be the main transport mechanism in cartilaginous fish and amphibians (29). In most other vertebrate species, an α-globulin was the major transport protein in mammals and a β-globulin mobility was found in birds, whereas in some species, radioactive vitamin D (metabolites) migrated with albumin mobility (29, 30). The first major link between GC and DBP was made by Daiger et al. Indeed, from the perfect parallelism between the electrophoretic mobility of GC (measured by immunologic techniques) and the mobility of [^14^C]vitamin D, they concluded that GC is the major transport protein for vitamin D (4). Peterson et al. had reported the purification of small amounts of human DBP and mentioned that GC was only a minor contaminant of “his” protein (31). However, the fact that the GC protein was identical to DBP was soon confirmed by three different laboratories reporting the purification of DBP from human serum, monitored by prior addition of [^3^H]25OHD (5–7). We saturated the binding capacity of human serum by adding, in addition to radiolabeled 25OHD, sufficient stable 25OHD to avoid elimination of apoprotein during the purification process, thereby achieving a higher recovery rate (5). All these groups were able to show that the protein they isolated based on its binding of [^3^H]25OHD was immunologically and electrophoretically identical to GC protein. The three groups initially applied different names, such as transcalciferin (5), but soon agreed upon DBP as abbreviation for the serum vitamin D binding protein (8). DBP was subsequently also purified from rat (32), chick (33) and many other species.

*In summary, GC was discovered in the early 1960s as a polymorphic serum protein and at the same time a serum protein (DBP) was identified transporting vitamin D and its metabolites. In 1985 and 1986 GC/DBP were found to be the same protein*.

## Gene Coding for GC/DBP

**Figure 2:**
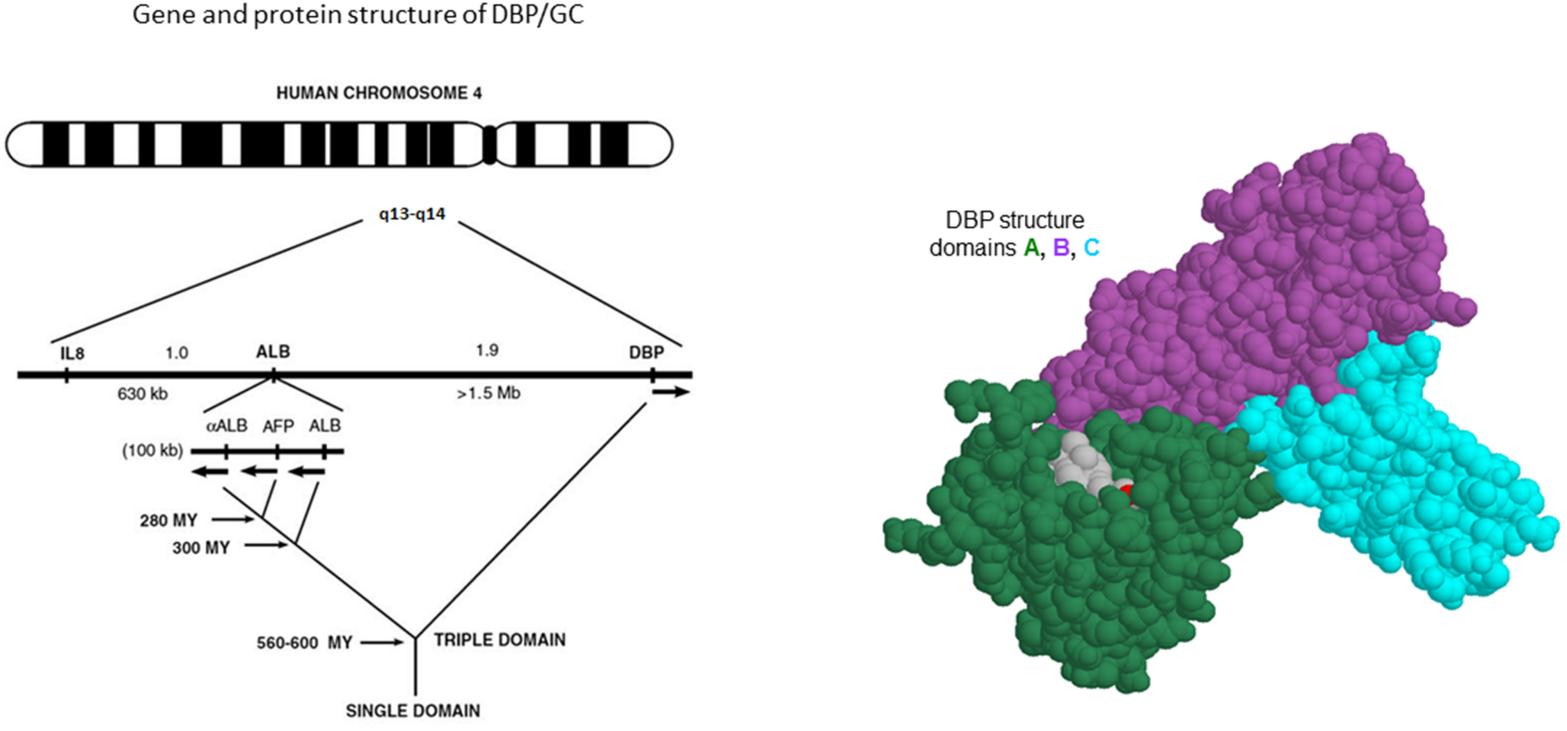
Gene and chromosome structure of GC/DBP and adjacent albuminoid family genes.

From early onwards, the three major forms of GC were shown to be the result of a pair of co-dominant autosomal alleles, resulting in homozygous GC1f-1f, GC1s-1s, GC2-2 or a mixture of these genes in most subjects. The gene for DBP/GC is localized on human chromosome 4q11-q13 as shown by *in situ* hybridization techniques, whereas the gene is localized on chromosome 5 or 13 in the mouse and rat, respectively (34). The gene is positioned close to the genes for albumin, α-fetoprotein and afamin (also known as a-albumin), with a centromere-DBP-albumin-α-fetoprotein-afamin-telomere orientation. Their protein products are mainly synthesized and secreted by hepatocytes. The DBP gene is also expressed in kidney, testis, endocrine pancreatic cells, and fat cells (35). Genetic analysis of the evolution of these sets of genes indicates that DBP might well be the oldest member of the family (Figure 2). Human and rat DBP have 13 introns and a 42 kb gene structure. The human gene codes for a 1690 nucleotide mRNA and a 458 amino acid long single chain protein, preceded by a 16 amino-acid signal propeptide.

*In summary, the structure of the GC/DBP protein was identified, located on human chromosome 4 close to the other members of the albumin gene family*.

## Evolution of Vertebrate GC/DBP

One of us (FS) studied the vertebrate GC gene and evolution of the predicted DBP protein primary structure, whereas another co-author (FR) evaluated the 3D structure of GC/DBP and its interaction with the vitamin D metabolites. The National Center for Biotechnology Information, U.S. National Library of Medicine (NCBI Gene) listed on June 7th 2019 GC gene sequences of 210 vertebrates (nine bony fishes, one amphibian, 14 reptiles, 59 birds and 127 mammals). In all clades, we ascertained the correctness of the searched gene not only by sequence homology but also by syntheny with its two neighboring genes, SLC4A4 and NPFFR2. A complete domain 1 primary structure (AA1-110) of DBP was found in 191 of these sequences. Here we summarize the essential conclusions, as a detailed analysis will be presented in a separate paper. First, in all major clades of vertebrates the predicted GC/DBP gene can be found, whereas [as expected from the literature (36)] the paralogous albumin gene is absent all fish, amphibia, lizards, snakes, and turtles. Second, by far the best conserved part of the primary structure of DBP (virtually 100% conserved in all species with a complete sequence) was a series of 28 cysteines that are ordered over the three protein domains as a typical repetition of “….C….CC….C….”. Two of these repetitions are present in the domain 1 primary structure. The evolutionary importance of cysteine pairs to form disulfide bridges in the primary structure was already suggested earlier, based on fewer gene and protein (six species for GC/DBP) structures (9, 37). Third, the highest protein homology was present in domain A, reflecting its crucial role to transport vitamin D and its metabolites, which are mainly hydrophobic. For instance, 25OHD_3_ has only two polar hydroxyl groups (on C3 and C25). These two critical recognition features involve in mammals residues Tyr-32 and Ser-76 to recognize the 25-OH and 3-OH moieties, respectively, which are located at two extreme ends of its ligand. Tyr-32 is not only well-conserved among mammalian species but also in bony fishes; however, the reptilian branch of vertebrates evolved to a different well-conserved asparagine. For residue 76, the conservation is the OH-group from either a serine or the slightly larger residue threonine. Moreover, amino-acid residues 8, 12, 24, 35, 68, and 107 that contribute to van der Waals interactions with vitamin D or its metabolites are also highly conserved among mammals and often substituted conservatively by other hydrophobic amino acids (Ile-12 or Val-12; Leu-107, or Met-107) that would form similar van der Walls interactions with the ligand. This conservative nature of the amino acids involved in the binding cleft of DBP explains the high affinity of DBP for 25OHD in all clades of vertebrates.

*In summary, GC/DBP is found in nearly all vertebrate species with well-conserved structure over the 500 million span of vertebrate evolution. This is especially the case for the ligand (25OHD) binding cleft*.

## DBP: Origin, Turnover, and Serum Measurements and Concentration

### Origin and Turnover

Vitamin D binding protein, as well as its close family members, is mainly produced in the liver, although the gene and protein are also expressed in very low concentrations in other tissues as mentioned above. The pool of DBP in rabbits is about 54 mg/kg with a half-life of 41 h and distributed between the intravascular pool (1/3) and the extravascular pool (2/3). The distribution is largely limited to the extracellular volume, in line with albumin (38). The half-life of DBP in human plasma is about 1.7 days and thus markedly shorter than the half-life of 25OHD, which is estimated at about 15 days, based on studies with deuterium labeled 25OHD in healthy subjects from the UK and Gambia (39). The estimated daily production of DBP is about 700–900 mg/d for an adult person (10 mg/kg/d). In comparison, the total body albumin is about 280 g for a normal adult and thus more than 300 times greater than that of DBP. About 40% of albumin is intravascular, with the remaining 60% distributed in the interstitial space of various organs (primarily muscle, adipose tissue, connective tissue, and skin) with an average interstitial concentration of about 60–70% of that of plasma. Rabbit and human data suggest a similar compartmentalization of DBP (38, 40). The absolute synthesis rate of albumin is about 150 mg/kg/day or 10.5 g/day for a 70 kg human. About 8.5% of plasma albumin and 4% of the total body albumin are synthesized each day, corresponding to a total body albumin turnover time of about 25 days or a half-life of 17.3 days. This is about 10 times longer than that of DBP but shorter than other circulating proteins (hemoglobin has a life span of 120 days) and similar to that of γ-globulins (41). The mRNA of DBP in liver is rather low (similar to the situation of albumin mRNA), suggesting a slow turnover of this mRNA comparing with the protein turnover. The clearance site of DBP is not fully understood, but DBP is partially filtered in the glomerulus and reabsorbed in the tubuli mediated by a carrier receptor mechanism (megalin, see below), followed by intracellular degradation. In rabbits, DBP holoprotein and apoprotein are cleared at about the same rate (42). The half-life of DBP is thus markedly shorter than the half-life of 25OHD and this implies that 25OHD is recirculated after degradation of DBP. Removal of sialic acid does not changes its plasma clearance (40).

### Protein Structure and Polymorphisms of DBP

**Figure 3:**
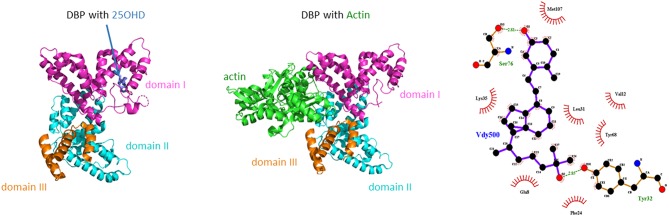
Crystal structure of human DBP in combination with 25OHD or actin. In addition the main amino acids involved in the binding of 25OHD to the cleft in the A domain of human DBP as shown.

The mature DBP structure of humans is 458 amino acids (AA) long, whereas rat, mouse, and rabbit DBP all are 460 AA long. DBP has a highly conserved number of cysteines and disulfide bridges. It has three domains, much like albumin, and these domains are probably the results of gene duplication of a singly common ancestor structure (34). Unexpectedly, the 3D structure of DBP differs substantially from that of albumin (20). The A domain has a cleft structure allowing to bind 25OHD with high affinity. The structure of the holoprotein (DBP plus 25OHD or analogs) confirms the predicted AA sequences (AA 35–49) responsible for binding of 25OHD and all other vitamin D metabolites. Indeed, there is only a single binding site for all D metabolites. DBP has the highest affinity for 25OHD-lactones, followed by 25OHD = 24,25(OH)_2_D > 1,25(OH)_2_D (5, 6, 43). The binding site of DBP for 25OHD is show in Figure 3. The structure of DBP with a vitamin D with a pentanor side chain modification, known from *in vitro* binding studies to have a high DBP affinity, allows to explain why such analog has a higher affinity (for detailed discussion see (20, 34). The binding site of vitamin D for DBP is totally different from that of the binding site of the vitamin D receptor (VDR) (44). The main characteristics of DBP are summarized in Table 2. Human DBP has an isoelectic point (IEP) of about 4.89, but this varies according to DBP/GC genotype. The stability of DBP at high temperature is markedly enhanced by binding to 25OHD. The holoprotein (DBP.25OHD complex) has a different IEP compared with the apoprotein, and this indicates that the protein undergoes a structural modification when bound to vitamin D metabolites (5, 45). DBP is highly polymorphic as it was originally discovered by this characteristic and therefore received its initial name of group-specific component. The three most common alleles and protein structures are shown in Figure 1. GC1 (1f or 1s) has a high degree (about 10–25%) of O-glycosylation in threonine position 436 with a linear trisaccharide (NeurNAc-Gal-GalNAC) whereas residue 434 is much less glycosylated (1–5%) by a disaccharide (without the final sialic acid). DBP/GC is similarly (poorly) glycosylated on AA 434 but not on AA 436 (being lysine rather than threonine) in DBP/GC1. The terminal sialic acid of DBP/GC 1 can be present or absent and therefore both DBP/GC1f and DBP/GC1s are present in serum in double bands with a very small difference in isoelectric point (Figure 1). Neuraminidase treatment can remove sialic acid and thereby eliminate this double band on isoelectric focusing (45, 46). The genetic or molecular (pre-or posttranslational) origin of the large number (>124) of variants of DBP in humans is largely unknown (34, 47) and the implication for the functions of DBP (see below) is unknown. The most common genetic variants (GC1s/1f/2) are due to polymorphisms in the third domain, whereas the few other variants are due to polymorphisms in the second domain [reviewed in (34)]. The best-known variant (GC1A1) is one found in Aboriginals and some South African blacks (48). Genetic polymorphism of DBP has also been documented in other species such as rats (32, 49), monkeys (50), swine, rabbits (24), chicks, and horses.

**Table 2:** Major characteristics of the human vitamin D binding protein.

Gene	- Located on chromosome 4q11–q13, close to albumin, α-fetoprotein, and afamin genes and in syntheny with its two neighboring genes, SLC4A4 and the neuropeptide receptor 2, NPFFR2 - Autosomal co-dominant gene transmission - Highly polymorphic gene/protein with more than 120 variants in human populations around the world. Polymorphisms also found in several other species including rodents
Protein	- 458 amino acids−58 kD–preceded by 16 amino acid signal propeptide - Three domains: a highly evolutionary conserved A domain with a cleft able to bind all vitamin D metabolites. The B and C domains can bind actin with high affinity - Single binding site at the surface of the A domain, creating a cleft for all vitamin D metabolites - Isoelectric point 4.6–5.0 depending on gene polymorphism or posttranslational modifications - Pool size: 2.8 g - T½: 1.7 days - Daily production: 10 mg/kg
Serum concentrations	- μmolar concentration (~200–600 mg/l) in normal adults - Mainly apoprotein configuration and <5% as holoprotein
Biological functions	- Binding/transport of all vitamin D metabolites with high affinity (25OHD lactone > 25OHD = 24,25(OH)_2_D = 25,26(OH)_2_D > 1,25(OH)_2_D > vitamin D, and vitamin D_3_ metabolites > vitamin D_2_ metabolites) - Binding of actin monomers and enhanced clearance of fibrillar actin - Binding of fatty acids and especially unsaturated fatty acids - Binding to megalin-cubilin receptor complex - Binding to membrane of leucocytes and activation of complement C5 system
Clinical implications	- DBP concentration and affinity define the free concentration of all vitamin D metabolites - Low concentration in fetus and neonates - Increased serum concentration when exposed to estrogens - Decreased serum concentration in case of liver diseases, nephrotic syndrome, malnutrition, severe acute trauma, or disease - Complete genetic absence leads to very low serum concentrations of all vitamin D metabolites without a clinical phenotype

### Measurement of DBP

DBP is usually measured by one of the many methods based on specific anti-DBP antibodies. This frequently requires species-specific antibodies when measured in different species. In the author's laboratory, polyclonal antibodies were generated for DBP from humans, rats, chicks, rabbits, mice, guinea pigs, and dogs. The binding capacity of serum for 25OHD can also be estimated by using radiolabeled 25OHD using a Scatchard plot to calculate its affinity and binding capacity. In most cases, the measured binding capacity was lower than the immunoassay values (51) but later technological improvements allowed to generate very similar concentrations between binding and immunoassays.

We used radial immunodiffusion as this avoids the need for major dilution of serum samples, but others used a wide variety of other assay methods, such as rocket electrophoresis, turbidimetry, or nephelometry, ELISA or even radioimmunoassay. More recently, DBP has also been measured by tandem mass spectroscopy after prior peptide digestion. This method requires specific synthesis of a mixture of labeled peptides common to all GC/DBP isoforms and in addition other peptides which are genotype-specific (21, 22, 52, 53). Based on a close collaboration between a team of the Leuven University, NIST (Karen Phinney and Lisa Kilpatrick) and Dr. Hoofnagle's group in Seattle, a reference standard for DBP (based on protein purified from homozygous GC1f, GC1s, and GC2 volunteers) and a reference method for an MS/MS based assay of DBP has been developed (53). An Elisa technique by R&D (54, 55) used monoclonal antibodies and was widely used to measure serum DBP concentration. The results obtained with this method surprised many experts as this assay showed race- and DBP/GC-specific results, whereby blacks or African-Americans (mostly having DBP/GC1f genotype) had markedly (−50%) lower serum DBP than whites (54). The authors, therefore, concluded that free 25OHD concentrations in African Americans were similar to those in Whites and that this could explain the paradox of “low” vitamin D status but solid bones and low fracture rate in African-Americans. These data and at least 50 other manuscripts using this assay all seemed to conclude that “we” all used wrong methods (total 25OHD) instead of free 25OHD to estimate the real vitamin D status (56). However, previous studies using polyclonal antibodies did not find racial differences in serum DBP (57). Extensive studies thereafter, using polyclonal and mass spectrometry based assays (22, 58, 59) all convincingly demonstrated that the R&D monoclonal DBP assay discriminates against DBP/GC 1f and that all results in genetically heterogeneous populations should be “retracted” or re-interpreted. The senior author of the Powe et al. paper later on agreed that the DBP results, based on the monoclonal R&D assay, from these studies were wrong (60).

The concentration of DBP in normal human serum is in the μmolar range (~6 μmol/l or about 300 ml/l) but different laboratories have reported mean concentrations varying between 200 and 600 mg/l. Most of these differences are probably due to lack of standardization of DBP assays and reference material. Hopefully, this will soon be corrected by using the NIST reference preparation (53). Most assays use polyclonal antibodies and therefore the effect of protein polymorphism in the final measurement is minimal. As mentioned above, one assay, however, used monoclonal antibodies and reported mean DBP concentrations that were very different according to the genetic (or racial) differences in DBP (54). Other monoclonal antibodies, however, do not discriminate GC/DBP isoforms (61, 62). When measured with a variety of polyclonal antibodies, DBP concentrations in humans with GC/DBP2 genotype are slightly (10–20%) lower than in GC/DBP 1 carriers. This was already know in 1966 and confirmed in many later studies [reviewed in (63)].

DBP circulates in serum in much higher concentrations than the combined concentration of all vitamin D metabolites, as serum DBP concentration is in the mid μmolar concentration whereas the serum concentration of the major metabolite, 25OHD, is usually below 100 nmol/l. This implies that <5, and usually <2% of DBP is a holoprotein (DBP plus vitamin D metabolite) and nearly all DBP circulates as apoprotein. This is quite different from the other binding proteins for ligands of nuclear receptors. The DBP gene transcription is regulated by a balance between hepatic nuclear factors (HNF) 1α and 1β, as is the case for other liver abundant proteins. However, unlike the regulation of albumin, HFN1α is a positive regulator and HFN1β is a dominant negative regulator of DBP expression (64). DNA methylation of the promotor region can also modify the DBP gene expression (65). DBP is already expressed in the yolk sac (as documented in animals) and later on in the fetal liver and thus appears in the fetal circulation. Indeed, DBP can be found in serum of human fetuses in the 3th trimester and its concentration in (human) cord serum is only about half that of maternal serum as reported by several authors (13, 34).

### Regulation by Hormones and Other Factors

The concentration of DBP in human serum is in the μmolar (micromolar) range as measured by immunoassay or, more recently, by MS/MS (34). In humans, exposure to estrogens increases serum DBP but androgens have no effects. Decreased serum DBP concentrations are found in patients with liver cirrhosis, malnutrition, peritoneal dialysis, and nephrotic syndrome (see also below DBP-megalin interaction). The concentration in the human fetus and cord serum is lower than in adult serum. Exposure to estrogens increases the serum concentration of DBP (e.g., due to intake of contraceptive estro-progestogens or during pregnancy). Therefore, the serum DBP concentration in pregnant women at the time of delivery is about twice the concentration found in cord serum (13, 14). Exposure to or loss of androgens does not change serum DBP concentrations in humans. Vitamin D deficiency or excess, or vitamin D resistance, idiopathic hypercalcemia of infancy, or osteoporosis and many other diseases (such as hyperparathyroidism or hyperthyroidism, sarcoidosis, cancer, Addison's disease, or growth hormone deficiency) have no effect on serum DBP concentration. DBP is slightly increased in acromegaly (66) and in some inflammatory diseases (such as in rheumatoid arthritis) (67). DBP concentrations are slightly decreased in serum of type 1 diabetes patients (68) and even more severely in BB or streptozotocin-induced diabetic rats (69), but better diabetes control can restore these concentrations (70). Decreased concentrations of DBP are also found in patients with a variety of kidney diseases or in case of renal loss of DBP. Patients with nephrotic syndrome therefore not only lose massive amounts of albumin but also large amounts of DBP (having a lower molecular weight than albumin). This urinary loss of DBP also entrails urinary loss of 25OHD and thus results in vitamin D deficiency (71). Low DBP concentrations are also found in animals or patients with genetic or acquired loss of megalin or cubilin, wo proteins functioning as cargo receptor to reabsorb serum proteins, filtered in the glomerulus and recovered in the renal tubuli (15) (see below). The genotype of DBP also has a small effect on the serum DBP concentrations as subjects with homozygous GC2-2 genotype have about 5–10% lower serum DBP concentrations compared to GC1 carriers (63). Whether this is due to a lower hepatic synthesis or more rapid clearance (lower glycosylation) is unknown.

DBP is a positive acute phase reactant after infections or minor trauma (72, 73). Severe trauma (e.g., hip fracture) or severe illness (such as patients requiring intensive care treatment) decreases serum DBP by more than 10%. Whether this is due to an increase in distribution volume, decreased synthesis or increased clearance as DBP or DBP-actin complex is not fully understood. Indeed, major tissue injury causes the release of intracellular actin into the blood stream, creating actin-DBP complexes that are rapidly cleared from the circulation (74), so that the positive effect of increased synthesis is largely compensated by even more rapid destruction and thus resulting an decreasing DBP concentrations. During ICU stay, serum DBP slowly increases and reaches normal levels again after about 10 days (Ingels et al., submitted). The serum concentration of DBP has a modest influence on the half-life of 25OHD as demonstrated by using deuterium-labeled 25OHD in healthy men living in the UK or The Gambia (39). This is not surprising as lower DBP concentrations, while serum 25OHD remaining similar, generate higher free 25OHD concentrations and a slightly higher catabolic rate. This study also showed that the half-life of 25OHD_2_ was slightly shorter than that of 25OHD_3_, in line with lower affinity of 25OHD_2_ for DBP.

### Evolutionary Aspects of DBP

Early in the evolution of vertebrates, after whole genome duplication, all elements of the vitamin D endocrine function became gradually operational (75). The vitamin D receptor, (VDR), coming from an ancestor nuclear receptor involved in detoxification, acquired a critical role in maintaining a normal calcium homeostasis and bone metabolism as to cope with an environment with lower access to calcium and a need for solid but light weighted bone in a terrestrial rather than an aquatic milieu. The CYP P450 gene family was broadened to include several genes with specific role in the activation and degradation of vitamin D (CYP2R1, CYP27B1, and CYP24A1). Finally, a specific transport protein for the lipid soluble vitamin D and its metabolites in serum is found in most fish and all terrestrial vertebrates studied so far. Not discussed here is the broad range of genes that are under the direct or indirect control of the vitamin D hormone (76) and which may explain why a poor vitamin D status not only results in a skeletal/calcium but also extra-skeletal phenotypes.

The VDR and the crucial CYPs for the metabolic activation of vitamin D are already present in cartilaginous fish. However, based on gel electrophoresis of radiolabeled 25OHD, Hay and Watson (29) concluded that the vitamin D metabolites in fish with a cartilaginous skeleton are transported by lipoproteins and not by a specific DBP as in higher vertebrates. Allewaert et al., however, found two vitamin D binding proteins in fish (carp) but only one resembles DBP (high 25OHD affinity and actin binding) (77). In amphibians (*n* = 12 species), Hay concluded that vitamin D was transported by lipoproteins. Allewaert, however, found in sera of amphibia (two rana species, Bufo marinus and salamandra) and reptiles, a 25OHD-binding protein with high affinity for 25OHD but with a low concentration (about 0.03 μmol/L or about 100-fold lower than in higher vertebrates) (78). DBP from reptiles is able to bind actin (as in higher vertebrates, see below), as revealed by sucrose gradient ultracentrifugation, but DBP from all amphibian binding proteins did not show actin binding. In reptiles (iguana, varanus, python, and geomyola), a DBP like protein able to bind 25OHD and actin was found as in chicks and mammals (78, 79). In turtle, trachemys scripta (a member of reptiles), DBP binds thyroxine as well as vitamin D (80). The situation in birds is complex as most species use a β-globulin, and a minority use a protein with an α-globulin or albumin mobility. DBP is present in bird serum and chick (gallus domesticus) but chick DBP has a slightly higher molecular weight than human DBP. Although avian DBP has a β-globulin mobility, it has a high degree of homology with human or rat DBP at protein and gene level (33).

Older studies, before the use of gene structure to define protein structures, revealed that mammals (65 species of 14 mammalian orders, including marsupials) use a transport protein with α-globulin (*n* = 65) or albumin mobility (*n* = 7 such as pacific dolphin or orcas, and three species of new world monkeys) (29). The DBP concentration in mammals (other than humans) are also in the μmolar range (as in birds). DBP levels are very low in rat fetuses and at birth. Thereafter, its concentration increases more than five-fold in adult rats. After rat “puberty,” androgens increase serum DBP whereas the DBP concentration does not change when rats are exposed to endogenous or exogenous estrogens (12). Vitamin D deficiency does not influence the concentration of DBP in rodents (nor in humans). Serum DBP concentrations in rats and mice increase when these rodents are exposed to androgens, contrary to what happens in humans and birds. Indeed, mouse DBP as measured by a mouse specific immunoassay, is higher in adult males compared to adult females. The DBP concentration did not markedly vary among different mouse strains [6–10 μmol/l in males and 5–9 μmol/l in females (81)]. Cortisol binding protein in rats, however, increases when animals are exposed to estrogens (as in humans). In guinea pigs, DBP concentrations decrease during pregnancy by nearly 50% (contrary to humans). Fetal and neonatal DBP concentrations of DBP are very low, similar to what has been observed in rats, despite the much greater maturity of guinea pigs at time of birth. Therefore, when comparing different species, we can conclude that DBP is already expressed early during fetal life but the DBP concentration at birth is much lower than later in life. Sex hormones change the concentration of DBP in all species and usually estrogens increase DBP concentrations except in rodents where androgens increase serum DBP.

DBP is found in all primates and migrates as an α-protein (as in humans), except in two New World strains. Their DBP is immunologically identical to that of other primates but has a different mobility. The genetic and molecular basis of this difference is not fully explained so far, as the molecular weight and isoelectric point are identical in these New World monkeys and humans (50).

The main lesson from all these studies is that vitamin D or its metabolites bind to lipoproteins in all vertebrates but from bony fish onwards, a protein of the albuminoid family, DBP, becomes the major transport protein with high affinity for 25OHD. The electrophoretic mobility of this protein however can vary from α- to β-globulin or albumin mobility. DBP concentrations are low in amphibia and reptiles (0.03 μmolar concentration) but are high in birds and mammals (~5 μmolar concentration).

*In summary, GC/DBP is mainly produced in the liver and circulates in high concentrations in serum of birds and mammals. It is usually measured by immune assays but now also by mass spectrometry. Its concentration in serum is stable and regulated by sex steroids. Decreased concentrations are found in liver or kidney diseases. Total (genetic) absence of DBP was so far only found in a single adult human without generating a bone or other clinical phenotype*.

## Functions of DBP (Table 2)

### Vitamin D Transport

#### DBP Is the Major Transport Protein for All Vitamin D Metabolites

**Figure 4:**
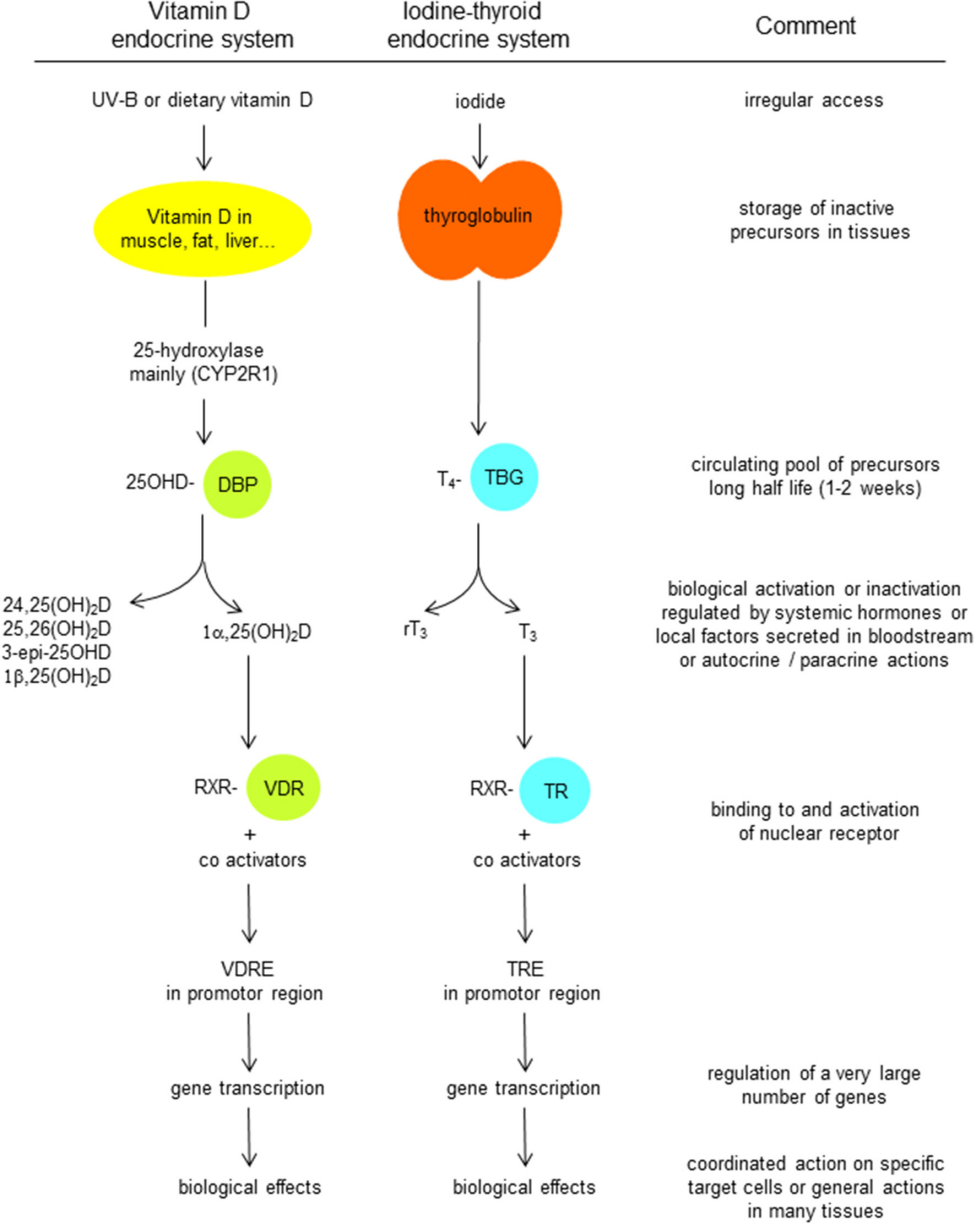
Comparison of the transport and metabolism of vitamin D endocrine system with that the iodine-thyroid system.

DBP, as its name suggests, is the major binding/transport protein for all vitamin D metabolites. There is only a single binding site in its A domain. Vitamin D is produced in the skin by photochemical transformation of 7-dehydrocholesterol (7DHC) into pre-vitamin D, followed by slow equilibrium with vitamin D itself. Vitamin D then binds to DBP and thereby drives the equilibrium in favor of vitamin D itself. This DBP-bound vitamin D is then rapidly taken up by the liver, and hydroxylated by CYP2R1 and possibly some other 25-hydroxylases, whereby the 25-hydroxylated metabolite is secreted into the circulation. It is then again, and now with higher affinity, bound to DBP. Vitamin D from dietary origin is absorbed in the intestine with about 70% efficacy in normal subjects. Although this process was for a long time considered to be a passive transport of a lipophilic molecule to access the lipid layer of the intestinal cells (82), more recent data suggest that vitamin D absorption and secretion from and into the lumen of the gut is medicated by carrier proteins that are also involved in cholesterol transport in the intestine (83). To what extent vitamin D esters are first digested and then absorbed or lost in the feces is still a point of discussion [see *Solanum malacoxylon* being a 1,25(OH)_2_D-glucuronide causing severe generalized calcinosis in grazing cattle in Argentina (84, 85)]. After passage thought the intestinal cell, vitamin D is transported by chylomicrons and thus uses the lymph pathway before it arrives in the general blood circulation (82). In that circulation, chylomicrons (including vitamin D) are mainly taken up by fat cells or by the liver, but there is also a gradual shift of vitamin D from chylomicrons to DBP. The metabolism and transport of vitamin D resembles the metabolic fate of iodine (also a nutritional product with only intermittent supply and essential for the generation of an essential hormone; Figure 4). In both situations, the prohormone, 25OHD, and thyroxin (T_4_) are bound with high affinity to a specific serum transport protein, DBP and thyroxin-binding globulin (TBG), respectively. Therefore, there is a large pool of circulating precursor with a long half-life (2 and 1 week, respectively), so that transient loss of supply of the nutrient (vitamin D or iodine) does not immediately cause lack of hormonal effect of the real ligand for their nuclear receptor [1,25(OH)_2_D and VDR, T_3_ and thyroid hormone receptor, respectively (Figure 4)]. The affinity of DBP for 25OHD is much higher than for the VDR, thereby keeping this precursor preferentially in the serum pool, and the same is true for T_4_. By contrast, the active hormones are ligands for their nuclear receptors to which they bind with high affinity, whereas their affinity for the serum binding protein is much lower. In consequence, the distribution volume of 25OHD is very close to that of albumin and DBP (~extracellular volume), whereas the distribution volume of 1,25(OH)_2_D and T_3_ is close to that of the intracellular volume.

Whether the role of DBP is essential for the overall metabolism and action of the vitamin D endocrine system can best be evaluated by data generated in animals or humans with total lack of DBP. Absence of other transport proteins for ligands of nuclear receptor are well-known and absence of TBG, cortisol, or sex hormone binding proteins does not create hormonal dysfunctions. This is explained by the very low total serum concentrations of the ligands for these binding proteins, so that the free (= not bound to their serum binding protein) hormone or hormone precursor is virtually normal. Therefore, the target tissues “see” normal free hormone concentrations (and in fact the concentration of these hormones are virtually normal when measured in tissues (86). Such data are the strongest arguments in favor of the free hormone hypothesis, posing it that the unbound ligands and not their total concentrations are physiologically important. For a long time, out of hundreds of thousands of human sera, not a single case of complete DBP deficiency was found, suggesting even that this protein might be essential for normal life. DBP null mice, however, did not have a phenotype and their concentrations of “total” 25OHD and 1,25(OH)_2_D were extremely low (close to the detection limit) (16). Nevertheless, such mice did not have rickets or another phenotype when kept on a vitamin D replete diet. In line with the role of DBP as responsible for generating a pool of circulating precursor, DBP null mice are much more prone to vitamin D deficiency when kept in conditions of vitamin D deficiency (no UVB light exposure and no vitamin D in their diet). In addition, these mice were more resistant to the toxic effect of exposure to high doses of vitamin D, probably by more rapid catabolism of vitamin D and its metabolites. In 2019, the first case of true total DBP deficiency was described in a 58-year old Canadian woman, with very low serum concentrations of 25OHD and 1,25(OH)_2_D and absence of serum DBP (all measured by gold standard technology of liquid chromatography-tandem mass spectrometry) (23). Nevertheless, no history of rickets or signs of osteomalacia were present and the patient had normal serum calcium and PTH concentrations. She was found to have a homozygous deletion of the complete GC gene region and part of the adjacent NPFFR2 gene. This latter gene codes for a GPCR involved in nociception and hypothalamic-pituitary axis (87). This rare event may be explained by parental consanguinity with Lebanese background. Both genes are known to be closely linked together during the whole evolution of vertebrates (see above in chapter on genetic evolution of GC/DBP). The sibling with only one mutated allele had about half of the normal DBP concentration (in line with the co-dominant inherence of both GC alleles) and serum 25OHD and 1,25(OH)_2_D concentration were well-below the normal concentrations in the healthy other sibling. The patient also had a debilitating ankylosing spondylitis, a known autoimmune disease. It is rather unlikely that this is related to the absence of DBP, as this phenotype is not found in DBP null mice. It may be related to the loss of the adjacent gene or due to repeated exposure to very high doses of vitamin D as to “correct her apparent vitamin D deficiency” although these interventions failed to increase serum vitamin D metabolites (23). Similarly, absence of albumin in humans is not causing a major phenotype.

#### Affinity of DBP for Vitamin D Metabolites

There is clearly only a single binding site for all vitamin D metabolites in DBP (Figure 3), in contrast to albumin which has several low affinity binding sites. 25OHD binds with high affinity but the absolute value differs slightly between species, and is dependent on the buffer medium, pH and temperature (88). Indeed, the affinity of DBP is higher (about 10 times) in barbital buffer at pH 8.6 compared to their affinity measured in different buffers at pH 7.4 (88). The affinity of DBP is highest for 25OHD-lactones, followed by about equal affinity for 25OHD and 24R,25(OH)_2_D or 25S,26(OH)_2_D. Its affinity for 1,25(OH)_2_D is about 10–100 lower compared to that of 25OHD. The lowest affinity is for vitamin D itself. The structure of the cleft on human DBP largely fits with these different affinities (20). Natural variants of the side chain such as in vitamin D_2_ and its metabolites may affect the binding for DBP. For humans and most mammals the difference in affinity is small, with about 20 % lower affinity for 25OHD_2_ compared to 25OHD_3_ (89, 90). In birds, DBP has a much lower affinity for 25OHD_2_ compared to 25OHD_3_ and this is supposed to be one of the main reasons for the low biological activity of vitamin D_2_ for the prevention of rickets in birds (91, 92). The relative difference in affinity between 25OHD_2_ and D_3_ was greater when measured in diluted serum (10-fold difference) (92) than when measured for purified chick DBP (three-fold difference) (33). Vitamin D_2_ is also largely ineffective in comparison with vitamin D_3_ to promote the intestinal absorption of calcium-47-isotope in Cebus Albrifons monkeys but for so far unknown reasons (93). In contrast to DBP, VDR does not discriminate between 1,25(OH)_2_D_3_ and 1,25(OH)_2_D_2_. Most enzymes involved in vitamin D metabolism also do not discriminate between D_2_ and D_3_ or its metabolites except for some non-CYP2R1 25-hydroxylases (94).

The affinity between DBP and its major metabolites has usually been studies by using [^3^H]labeled vitamin D metabolites and either diluted serum or purified DBP. We also estimated the affinity (Ka) in rat serum as the ratio of “on rate” over “off rate” by measuring the association and dissociation rate constant (95). The dissociation rate constant was highly temperature dependent, being about 1 day at 4°C and between 2 and 12 min at 37 and 24°C, respectively. The half-association time was about 1 min at 4°C and even more rapid at higher temperature. The best estimation of the Ka based on these rate constants was very similar to the Ka measured by direct binding assay, charcoal precipitation of free ligands and Scatchard plot analysis. As the affinity is dependent on temperature, pH, buffer conditions, and methodology, variable data have been generated but there is general agreement that human DBP has a very high affinity for 25OHD, with a Ka of around 1.5 × 10^8^ M^−1^ (34, 88) at 37°C. Whether the different isoforms of DBP have different affinities for 25OHD is a matter of debate as one group (96) concluded that DBP/GC2 has only half of the affinity of DBP/GC2s and this isoform had again only half the affinity of DBP/GC1f. Three other laboratories, however, independently, could not find significant differences in affinity for 25OHD when measured by Scatchard plot using highly labeled [^3^H]25OHD rather that vitamin D_3_ itself used by Arnaud and Constans (34, 60). When we compared the affinity of 20 human samples, only a very minor difference in affinity was found with a Ka of 1.63 (±0.29) × 10^−10^ M for GC1s homozygotes compared to 2.11 (±0.25) × 10^−10^ M for GC1f, and intermediate values for DBP/GC2 homozygotes (79). The rank order of these relatively small differences was, moreover, different when the measurements were done in a phosphate buffer compared to a Hartman buffer (both at pH 7.4) (33). A rare variant of DBP/GC (GC Aborigine or GC1A1) as found in Aboriginals and some blacks from South Africa (48) have similar Ka values (88). The affinity of DBP from different species as measured in the author's laboratory is shown in Table 3. The highest affinity for 25OHD is found in serum from rats [confirmed by (49)] and 5 species of amphibians (79). Lowering the pH rapidly decreased the affinity (to very low levels at pH 5), but the affinity increased at pH 8–10 with the highest values at about pH 8.6 (about 10-fold higher in mammalian DBP but not in DBP from chicks or toads). The affinity of DBP for 1,25(OH)_2_D is about 1.5 × 10^7^ M^−1^, with little difference between DBP/GC genotypes and only a minor increase in affinity at higher pH (88). The affinity of purified DBP from human, rat, and chick serum was very similar to that measured in (highly) diluted serum. The variation of the assay results are about 25% of the prefix of the Ka value. When comparing methods to separate bound and free, charcoal or filter assays generated similar results (67, 88, 97). The affinity of DBP for 1,25(OH)_2_D is about 30–50-fold lower than for 25OHD in all species studied so far. The Ka at pH 7.4 and 4°C is about 1-5-2 × 10^7^ M^−1^ in most species but about 10 times higher values were measured in diluted rat (49, 88) and amphibian sera (Table 3). In chicks (*Galllus domesticus*), the Ka of DBP for 25OHD at 4°C and pH 7.4 is 1 × 10^9^ M^−1^ (33). Bouillon et al. could not find a specific vitamin D3-binding protein as described before by Edelstein et al. (33, 98). Indeed, labeled vitamin D behaved similarly to labeled 25OHD on simple and crossed immunoelectrophoresis of chick DBP. In DBP-free serum, both metabolites were bound to albumin.

**Table 3:** Affinity of DBP from different species for the major vitamin D metabolites^*^.

**Species**	**25OHD**	**1,25(OH)_**2**_D**	**Comments**
Human	5 × 10^8^ M^−1^	1.5 × 10^7^ M^−1^	Variation between 3 and 10 × 10^8^, depending on buffer and DBP/genotype
Monkeys	5 × 10^8^ M^−1^	1.5 × 10^7^ M^−1^**^^	Measured in old and new world monkeys. In *Cebus albicans*, DBP migrates with albumin mobility but has a similar Ka
Rat	6 × 10^8^ M^−1^	1.4 × 10^8^	Measured in three strains
Rabbit	1.5 × 10^9^ M^−1^	NM	
Chick	4 × 10^8^ M^−1^	1.7 × 10^7^ M^−1^	
Reptiles	NM	NM	
Amphibia	2–14 × 10^10^ M^−1^	2.6 × 10^8^M^−1^	Measured in 5 species

* *Measured in diluted serum at pH 7.4 and 4°C, unless specified otherwise; all values are expressed as Ka at M^−1^ values*.

** *The Ka for 1,25(OH)_2_D varied between 1.2 and 2.6 × 10^7^, depending on buffer and DBP/GC genotype*.

*NM, not measured*.

#### DBP and Concentration of Unbound Vitamin D Metabolites: Free or Bioavailable D Metabolites

As the DBP has a high affinity for most vitamin D metabolites, and also has a much higher molar concentration compared to its ligands, the free concentration of all its metabolites is very low, in fact much lower than that of other ligands of nuclear receptors. The free concentration of 25OHD and 1,25(OH)_2_D were first calculated^1^ based on the measurements of total concentrations of the metabolites, total concentration of DBP, the best estimation of the affinity and the law of mass action (13, 14). Free hormones such as cortisol, thyroxin, and sex hormones are usually a better marker of their biological activity than their total concentrations (101). These free hormone concentrations are also better feedback-controlled than the total hormone concentrations. The best example is that of extreme deficiency of transport proteins of one of these ligands, resulting in a dramatic decrease in total but keeping free hormones within the normal range. Is this also applicable to the major vitamin D metabolites? Two different terminologies are frequently used for free hormones: some define free hormone as non-protein (either to the specific binding protein and albumin) bound hormone, whereas bioavailable hormone is the combination of free and loosely albumin-bound hormone.

**Figure 5:**
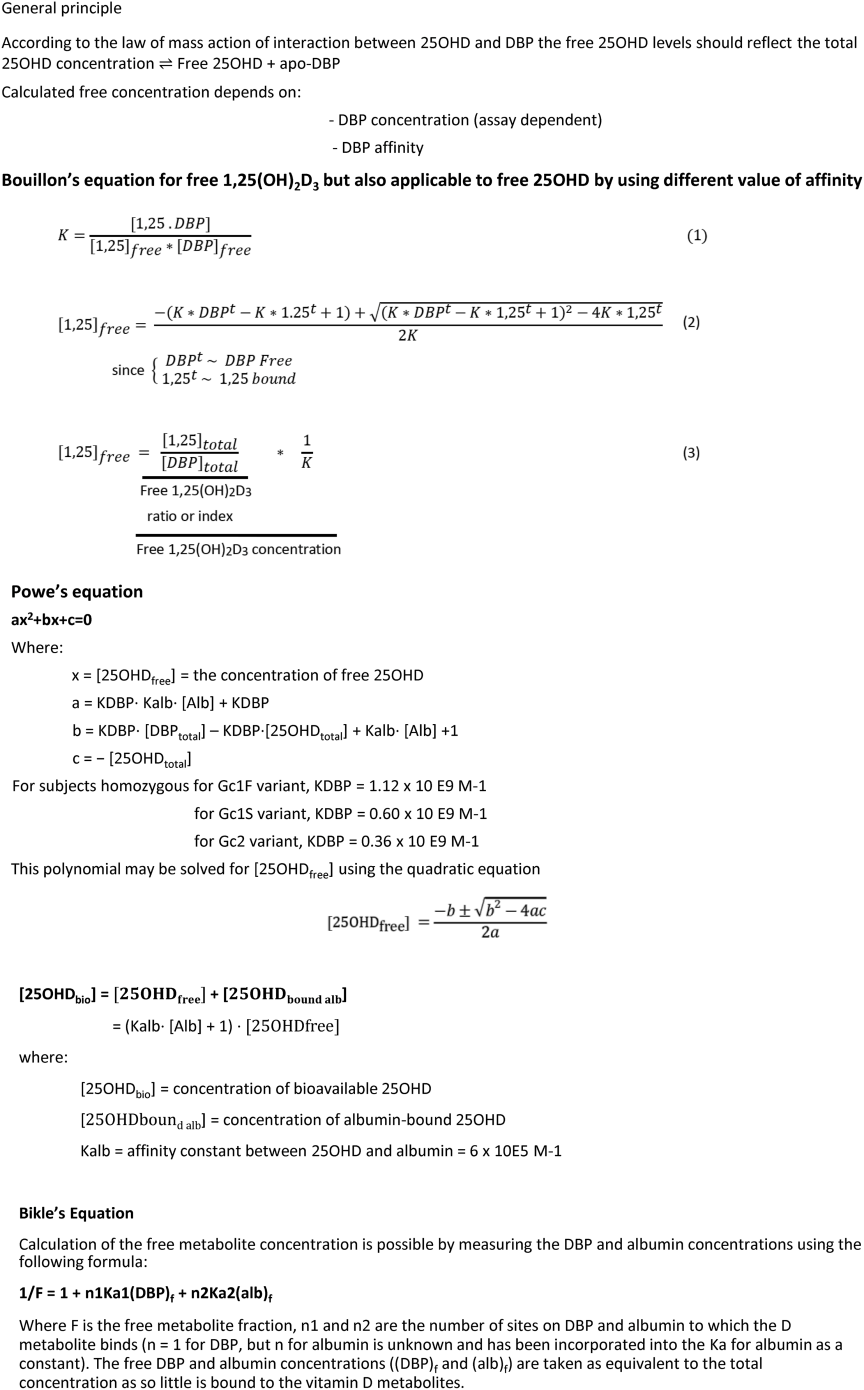
Formulae to calculate the free concentration of 25OHD or 1,25(OH)_2_D (14, 43, 54).

The first major difference with other transport proteins is the combination of high affinity and very high concentration of DBP so that the free concentration of 25OHD and 1,25(OH)_2_D are very low in absolute and relative concentrations. The free 25OHD and 1,25(OH)_2_D concentrations are about 10 and 1 pmol/l (14). This also means that the free 25OHD concentration is <0.1% of total 25OHD and that free 1,25(OH)_2_D is about 1% of its total concentration. Due to difference in affinity, the molar ratio of total 25OHD over total 1,25(OH)_2_D is about 500 but free 25OHD is only 10 times higher than free 1,25(OH)_2_D. In Figure 5, a few formulae used to calculate the concentration of free vitamin D metabolites are shown. All formulae use the law of mass action as published by Vermeulen et al. (99) (see also footnote 1) for androgens and by Coolens et al. (100) for cortisol. The simplest way to express free hormone (vitamin metabolite) concentration is a calculation of the free hormone/metabolite as a molar ratio of molar concentration of hormone/metabolite over molar concentration of its binding protein (DBP). As DBP has such a high capacity, the concentration of the apoprotein is indeed virtually also close to that of the total DBP concentration. Such molar ratio of 1,25(OH)_2_D:DBP is nearly identical (*r* > 0.95) to that of calculated free 1,25(OH)_2_D (14).

Free 25OHD is not feedback-regulated in humans nor in animals. Increased intake of vitamin D or higher sun exposure increase total as well as free 25OHD concentrations, and vitamin D deficiency does not change DBP concentrations whereby total and free 25OHD concentrations decrease to about the same degree. In most studies, increase in DBP by estrogen intake does not increase serum 25OHD (14, 34). Whether free 25OHD is a better predictor than total 25OHD for health outcomes is controversial and will be discussed elsewhere in this Journal and has recently been reviewed several times (34, 60). Free 25OHD can be calculated based on DBP concentrations and the affinity of DBP for 25OHD but the DBP assay is not standardized yet and the exact value of the Ka value at 37°C is also not perfectly known (see above). Therefore, the predictive value of calculated free 25OHD is yet unknown. Free 25OHD can also be directly measured by ultrafiltration or dialysis (43, 102) or by ELISA (103). Dialysis and ultrafiltration are technically difficult and face problems due to interference of adsorption of very low vitamin D metabolites to glass or plastic tubes, and even a minute impurity of labeled metabolites represent concentrations potentially higher than the free metabolites. In normal subjects, the directly measured free 25OHD concentration has a strong correlation with the calculated free concentrations (taking into account a variation coefficient of about 10% of all measurements). In the MrOs study on elderly men, calculated free 25OHD (using polyclonal DBP assays) correlated strongly with directly measured free 25OHD (*r* = 0.80–0.83) (22, 59). Moreover, the calculated or measured free 25OHD concentration strongly depended on total 25OHD (*r* = 0.96 and 0.83, respectively). Of course, as for other free hormones, the direct measurements are especially important in case of abnormal concentrations or affinity of the binding proteins. This aspect has not yet been fully evaluated for free 25OHD. Free 1,25(OH)_2_D concentration probably “behaves” more like free thyroxin, cortisol or sex steroid hormones. Indeed, the total 1,25(OH)_2_D concentration correlates with DBP concentrations in most studies whereas this is not the case for total 25OHD. Increased DBP by estrogen exposure or early pregnancy increases DBP and total 1,25(OH)_2_D very similarly, so that the free concentrations remain unchanged (14). Similarly, in sexually mature hens, DBP and 1,25(OH)_2_D increase several fold but 1,25(OH)_2_D increases more than the DBP so that the free 1,25(OH)_2_D concentration is high, in line with the high calcium demands for egg shell calcification. When, however, egg shell calcification is blocked by a thread in the uterus, soft eggs are produced, calcium requirements drop, and total and free 1,25(OH)_2_D fall back to the level of immature chicks (104). Infusion of large amounts of human DBP in blood of normal rats, increases combined rat and human DBP concentrations and also increases total 1,25(OH)_2_D concentrations (67). Total absence of DBP in mice results in very low (nearly undetectable) total 1,25(OH)_2_D concentrations but the tissue concentration of free 1,25(OH)_2_D is normal (86) and serum calcium and PTH status remain normal. In the only human case of biallelic mutation of the DBP/GC gene, resulting in undetectable serum DBP concentrations, total 1,25(OH)_2_D was nearly undetectable but without consequences for calcium or PTH homeostasis (23). Also animal studies came to the same conclusion as rabbits immunized against 1,25(OH)_2_D-conjugates as to induce antibodies for immunoassays have a more than 100-fold increase in total serum 1,25(OH)_2_D concentrations without repercussions on calcium homeostasis (51). There are at present no publications reporting direct measurements of free 1,25(OH)_2_D. Whether free 1,25(OH)_2_D is a better predictor than total 1,25(OH)_2_D concentrations for health outcomes is unclear. In the MrOs study, free 1,25(OH)_2_D concentrations were a better predictor of inflammatory markers than total 25OHD, total 1,25(OH)_2_D or free 25OHD (105). As the concentration of DBP in maternal serum (at time of delivery) is about twice the concentration of DBP in cord serum, the maternal:neonatal ratio of DBP and vitamin D metabolites may be informative for the relative importance of free vs. total vitamin D metabolite concentrations. From previous genetic studies, it is well-documented that there is no transplacental transfer of DBP/GC (24). The total and free 25OHD concentrations show a high correlation between the maternal and fetal compartments, with *r* values usually above 0.6. The total 25OHD concentration is much higher in the mother than in the neonate but the free 25OHD is higher in cord serum. This probably reflects a good transplacental transport and a contribution of fetal 25-hydroxylase activity to the fetal vitamin D status. The correlation of total and free 1,25(OH)_2_D between both compartments is somewhat lower than for 25OHD and the free 1,25(OH)_2_D concentration in the fetus is slightly higher than in maternal serum. As fetal (and placental) 1,25(OH)_2_D production may be partly responsible for the higher neonatal ionized concentration (and placental calcium transport), as shown in sheep by Care (106), such higher fetal 1,25(OH)_2_D may reflect its biological role.

**Figure 6:**
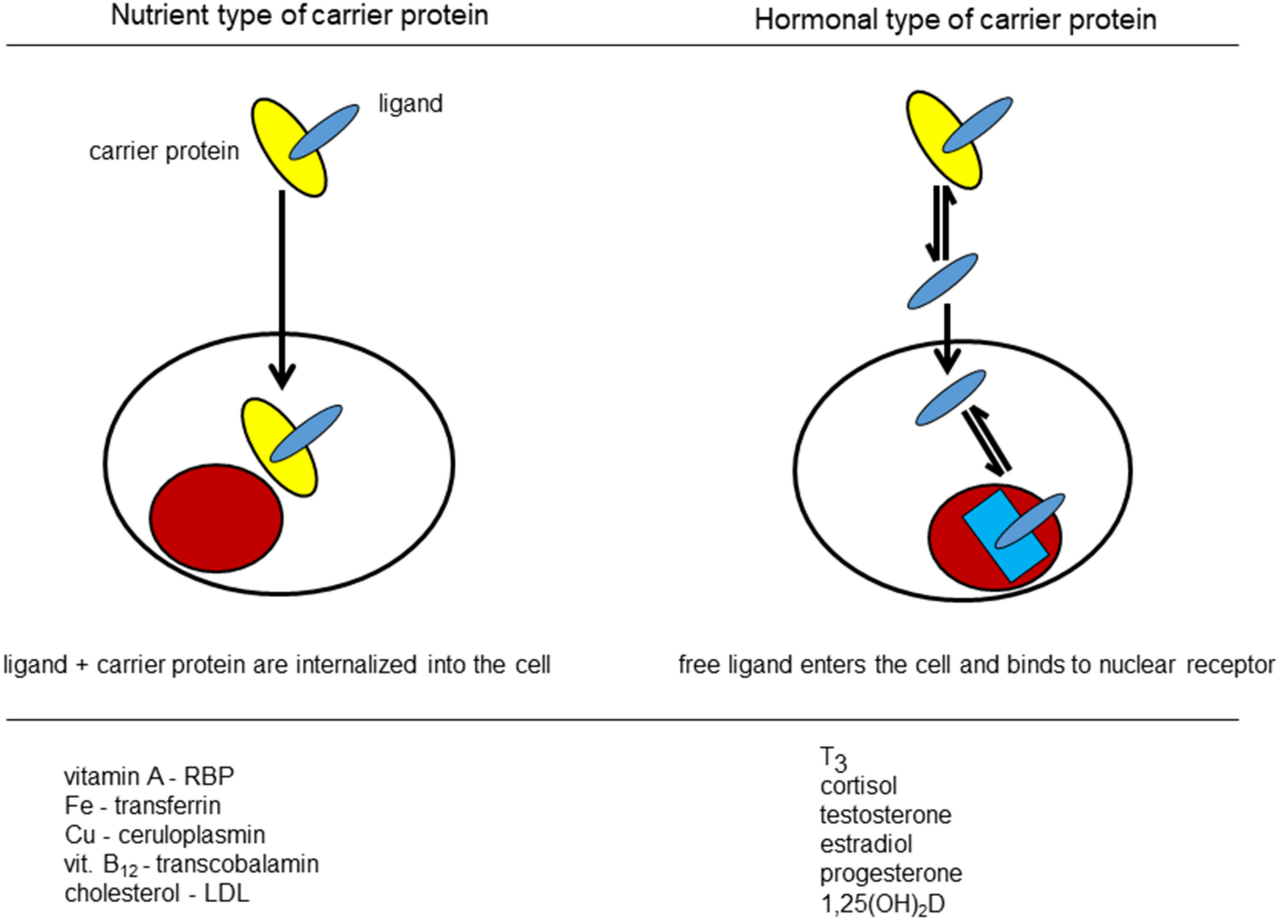
Model of nutrient and hormonal type of access of extracellular ligands to cells.

The most important implication of the free hormone hypothesis is that only the free or bioavailable hormone/ligand/vitamin D metabolite has access to the cytoplasm or nucleus of the cell. Indeed, cells have access to the total concentration of ligands bound to serum proteins when the cell uses specific receptors to internalize these complexes (Figure 6). As example, cholesterol carried by lipoproteins can be internalized by hepatocytes or other cells using specific LDL or HDL receptors and, therefore, these cells have access to the total concentration of cholesterol. “Free” or unbound cholesterol” is not discussed or used for clinical purposes. Other examples are shown in Figure 6. By contrast, most hormones enter cells by diffusion and only the unbound hormone enters most cells. This has clinical implications, as free hormone measurements are the preferred technique to estimate thyroid function. The real situation is probably slightly more complex as excess free ligands in the “nutrient type” of cellular import may influence cell function (e.g., iron overload). If cells express receptors for some binding proteins such as receptors for the sex steroid-binding protein or the presence of megalin in some cells, then such cell membrane receptors allow the import of the binding proteins (e.g., DBP) with all its ligands (see below). For some hormones, specific membrane carriers (such as membrane transporters for thyroid hormones) further complicate the model. For most situations, however, the free hormone model is physiologically and clinically relevant. We think that the DBP*1,25(OH)_2_D complex belongs to the hormonal type of serum transporters as only free 1,25(OH)_2_D influences cell function. Its free concentration in the picomolar range comes close to the affinity of VDR for its major ligand. For 25OHD, the question is different and highly relevant. Indeed, the free 25OHD concentration is around 10 pmol/l and this seems low in comparison with the affinity of the major CYPs responsible for its further metabolic activation. This raised the question whether (free) 25OHD has real access to CYP27B1 or CYP24A1 in many cells and thereby has functional implications. The role of megalin in the kidney tubuli will be discussed below (chapter “DBP binds megalin”). Most other cells do not express or have only minimal expression of megalin. Probably the best method to explore the functional role of 25OHD in the local production of 1,25(OH)_2_D can be found by deletion or overexpression of CYPs (CYP27B1 or CYP24A1) in specific tissues and expose such mice/animals to variable serum concentrations of 25OHD and or DBP. Tissue specific deletion of CYP24A1 in mammary cells had a clear effect on the risk of breast cancer (107). Overexpression of CYP27B1 in bone cells seemed to be beneficial for bone (108, 109) and deletion of this gene in growth plate chondrocytes generated bone effects as long as the growth plate remained fully active (110). Therefore, from these few examples, it seems that 25OHD has sufficient access to some target cells and generate local actions without full understanding how such low free or bioavailable concentrations can activate these genes and cells.

When comparing four different species (humans, rats, guinea pigs, and sheep) and calculating free 1,25(OH)_2_D based on total serum 1,25(OH)_2_D and DBP concentrations in the maternal and neonatal compartments, free 1,25(OH)_2_D is higher in the fetus/neonate than in their mothers. The concentrations of total and free 1,25(OH)_2_D concentrations show a good correlation in all these species (111) so that it is likely that the fetal production of 1,25(OH)_2_D contributes to this gradient and maybe also to the greater concentration of calcium in the fetus compared to the maternal calcium concentrations. Whether bioavailable (= sum of free and ligands loosely bound to albumin) is more important than free ligands is not fully understood and has been discussed but not “solved” in other reviews.

Whether the free hormone hypothesis also applies to the vitamin D endocrine system can be demonstrated by *in vivo* data as summarized above but also by dedicated *in vitro* studies. Addition of purified DBP or serum to culture media followed by measuring the biological response of cells or tissue to either 25OHD or 1,25(OH)_2_D has repeatedly shown that the presence of DBP impairs the biological action, whether using the expression of a natural gene/protein or using a genetically engineered gene construct. As examples:

1. The proliferation of human lymphocytes, cultured in a DBP free medium, can be inhibited by 50% by 1,25(OH)_2_D at 10^10−12^ concentrations, but required 100-fold higher molar concentrations if purified DBP is added at the physiologic concentration of 5 μmol/l (112). DBP counteracted the action of several vitamin D analogs with the same rank order as predicted on the basis of their affinity for DBP.
2. Resorption (measured by release of radioactive calcium) of bone tissue explants (forelimb bones from 18d old fetal rats, *in vivo* exposed to radioactive calcium) cultured in the absence of DBP is stimulated by low concentrations of 1,25(OH)_2_D. Adding purified rat or human DBP (5 μmol/l) impaired bone resorption but rat DBP was much more potent, in line with its 6–10-fold higher affinity for 1,25(OH)_2_D (113).
3. Cathelicidin, a well-known target of the vitamin D hormone, is stimulated by 1,25(OH)_2_D or 25OHD in human monocytes, cultured in serum from DBP null mice. Adding serum from normal mice however impaired this effect. The authors did not observe a megalin-mediated action (see below) (114).
4. Human foreskin keratinocytes express mRNA for CYP24A1 when exposed to 1,25(OH)_2_D. This action was markedly inhibited by culturing these cells in the presence of diluted serum compared to an albumin only based medium. Similarly, the production of radioactive 24,25(OH)_2_D from [^3^H]25OHD was inhibited by DBP (115).
5. Dermal fibroblasts from a patient with biallelic mutations of the DBP/GC gene or from normal volunteers were exposed to exogenous 25OHD, either in the presence of 10% normal serum containing DBP or 10% DBP deficient serum. The uptake of 25OHD in all cells was markedly inhibited by the presence of human DBP (23).

All these experiments clearly demonstrate that the presence of DBP decreased the access and actions of vitamin D metabolites in a wide variety of cells.

### DBP Binds to Megalin-Cubilin Receptor

Patients with nephrotic syndrome loose massive amounts of serum proteins in their urine, exceeding the liver capacity to replace the daily loss. DBP has a smaller molecular weight than albumin and is lost together with 25OHD and other vitamin D metabolites. This renal loss of 25OHD frequently results in a poor vitamin D status as first reported in 1977 (71) and subsequently confirmed in many other studies. Megalin (global or kidney-specific) knockout mice are unable to reabsorb several proteins in the renal tubuli as it is a cargo receptor for several serum proteins, allowing to prevent their urinary loss (15). Megalin is a membrane receptor and requires cubilin as co-receptor (116), and both are expressed in several tissues but mainly in the luminal brush border membrane of the renal tubuli. Megalin is a giant protein of 600 kDalton and belong to a family of low-density lipoprotein receptors. Therefore, it is also known as LRP2. This protein was initially identified as autoantigen in an experimental model of membranous nephropathy, called Heymann nephritis (117). Megalin is a well-conserved gene/protein, already present in nematodes. In mammals, the expression of LRP2/megalin is restricted to specialized absorptive epithelia in the brain, the eye, the lung, the kidney, and the reproductive tissue. Therefore, the major phenotype of a global megalin knockout is characterized by major developmental brain abnormalities, usually even lethal. Megalin binds and internalizes a large number of low molecular weight proteins, responsible for transporting a variety of lipids, vitamins, and hormones. This includes DBP, the retinol-binding protein, and transcobalamin. Thereby, megalin-mediated clearance of these binding proteins from the tubular lumen serves to retrieve essential vitamins and hormones bound to these carriers and to prevent uncontrolled loss of essential metabolites by glomerular filtration. More than 20 other proteins, including thyroglobulin (118) are also known to be ligands of these membrane receptors (119). In the absence of one of these co-receptors (megalin or cubilin), many low molecular weight proteins are lost in the urine, known as tubular proteinuria. Loss of these receptors can be due to a variety of acute, chronic or genetic diseases. In such diseases, DBP is also lost in the urine together with 25OHD and this loss of vitamin D metabolites may exceed the daily supply and synthesis of vitamin D and therefore cause vitamin D deficiency and rickets (15). This also implies that the renal tubuli, where CYP27B1 is transforming 25OHD into 1,25(OH)_2_D, has access to 25OHD by either the serosal site (and thus mainly access to non-DBP bound 25OHD), or via the luminal site (where DBP in complex with 25OHD is internalized by the megalin-cubilin receptor complex). Thereafter, reabsorbed proteins are broken down in lysosomes, leaving the luminal 25OHD available for metabolic activation. Megalin is then recycled to the luminal membrane. This phenomenon does not imply that the megalin pathway is essential [as suggested by some expert reviewers (120)] for the renal synthesis of 1,25(OH)_2_D, as megalin-deficient mice do not develop rickets if the urinary loss of vitamin D metabolites is compensated by sufficient intake. Neither do DBP-deficient mice develop rickets if they have access to sufficient vitamin D. Because the megalin receptor is a cargo receptor for a large number of proteins, loss of one of these membrane receptors can cause a variety of deficiencies, apart from vitamin D deficiency. Whether megalin- or cubilin-mediated uptake of DBP is also operational in other cells, such as placenta or parathyroid gland is insufficiently documented so far. However, the gene expression of megalin outside the kidney, brain, and eyes, is usually very low. The lung may be an exception and is now known to be an important target for vitamin D action (121). As discussed above, such transport mechanism, if operational outside the kidney, would expose cells expressing these cargo receptors to DBP bound 25OHD rather than free 25OHD.

### DBP Binds Actin

About 50 years ago, three major binding proteins were known, the serum binding protein, now identified as DBP/GC, the intracellular/nuclear VDR (now known to be a member of the nuclear transcription factors), and an intracellular 6S binding protein (MW about 90,000 Daltons), with preferential binding for 25OHD and which was considered as a cellular “receptor” for 25OHD (122, 123). This protein was found in most tissues but also in whey and milk of humans and mammals. In 1977, however, we demonstrated that the 6S 25OHD-binding protein was in fact a complex of DBP with an ubiquitous cytosolic heat-labile compound that was by itself unable to bind vitamin D metabolites (11). This phenomenon was confirmed by using antibodies against DBP (11, 124). A few years later, while trying to identify this intracellular compound by adding different proteolytic enzymes and DNAase, Van Baelen et al. (125), found that the addition of DNAase created a triple complex DBP-unknown intracellular protein-DNAase. Based on this observation, the Leuven group identified actin as the cytosolic protein that binds with high affinity to create the so-called intracellular 25OHD-binding protein/receptor. Actin-DBP binding does not involve the presence or absence of vitamin D ligands, or vice versa. This was confirmed by resolving the 3-dimentional structure of DBP-actin by three different groups (126–128).

Many actin-binding proteins regulate or control intracellular actin polymerization or disassembly, including profilin (129). The extracellular binding proteins are, however, mainly DBP and gelsolin (also known as brevin). Profilin in also present in platelets and can act as an actin sequestrant but with a 1,000-fold lower affinity and therefore of probably minor extracellular importance (130). Gelsolin-actin forms a 2-1 complex as gelsolin is able to sever actin polymers and then one gelsolin binds and caps each filament fragment. Together with profilin and gelsolin, DBP creates a well-coordinated strategy with complementary mode of action as to rapidly remove actin (polymers). These observations have several implications. First, DBP is not found inside cells when plasma contamination is carefully avoided during tissue preparation. Thus, it is rather unlikely that extracellular DBP interferes with the key functions of intracellular actin. Second, actin-DBP complexes only happens when intracellular actin is released in the bloodstream. Indeed, Van Baelen et al. (125) clearly showed that DBP was able to depolymerize polymeric actin. Actin can easily switch from monomeric into polymeric actin in the cytosolic milieu, thereby helping in building the intracellular organizations of cells. In the plasma milieu, actin monomers are rapidly transformed into polymeric structures, which could result in clogging the microcirculation much like fibrinogen/fibrin. This process could be accelerated by the aggregation of platelets as actin-ADP binds to platelet surfaces and acts as agonist for platelet aggregation (131). DBP, however, is able to build a one-to-one complex with actin and together with other actin severing/binding proteins (e.g., brevin) in serum, allows to prevent the formulation of actin polymers in the circulation (74, 125). These proteins have different roles in actin depolymerization by either severing or capping the actin chains and creating actin-DBP complexes that are more rapidly cleared from the circulation that DBP alone (74). Actin and DBP are very tightly bound (Ka of ~2–5 × 10^8^ M^−1^) (125, 132) so that these complexes are de facto not dissociated once formed in the circulation. However, actin-DBP is rapidly cleared from the circulation with a half-life of <1 h in rabbits (74). The damage caused by actin polymerization has been directly demonstrated in DBP null mice (133). Actin infusion in such mice resulted in more severe acute lung inflammation (vascular leakage, hemorrhage, and thickening of the vascular wall) compared with normal mice. This was confirmed *in vitro* as lung endothelial cells, exposed to DBP-actin complexes generated enhanced cell death (133). Third, the 3-D structure shows all three domains of DBP being in direct contact with actin and interacting through a variety of contact mechanisms. The substantial surface areas from both DBP and actin that are involved in intimate protein-protein contacts explains the high affinity interaction between these two proteins. Moreover, the DBP residues involved in actin binding are less well-conserved between all species with known DBP structures than the vitamin D-binding domain. The contact surface is greater than the contact surface of actin with two other actin-binding proteins (gelsolin and profilin) together (126–128). These data confirm the actin-scavenger role of DBP in higher vertebrates. However, DBP from some fish and amphibians are unable to bind to actin and create an actin-DBP complex. The molecular reasons for that absence of actin interaction are so far unknown. Fourth, DBP-actin interaction can be used to isolate DBP from several species, as actin can be used for affinity chromatography when no species-specific antibodies against DBP are available. In clinical or research situations, the presence of actin-DBP complexes in serum or the (low) DBP concentration in serum can be used a marker for the disease severity and has prognostic significance in patients with severe illness (134). The greatest decrease in serum DBP has been found in patients with acute/fulminant hepatic necrosis (whereby serum DBP may fall to about 10% of normal values) rhabdomyolysis, sepsis, or major traumas (135–137).

### DBP Binds Fatty Acids

Triglycerides are bound in serum to several lipoproteins whereas free fatty acids are mainly loosely bound to albumin. However, all members of the albuminoid family are able to bind fatty acids. DBP binds to fatty acids present in membrane phospholipids (see DBP and inflammation) of leucocytes but also bind fatty acids, and especially poly-unsaturated fatty acids (16:1, 18:1, 18:2, and 20:4) are associated with DBP/GC as measured by gas chromatography (17). When [^3^H]arachidonic acid was added to serum about 75% was found to be bound to DBP. The molar ratio of fatty acids/DBP is about 0.4 compared to 1.8 for albumin. Mono- and polyunsaturated fatty acids impair the binding of 25OHD and 1,25(OH)_2_D to DBP. Indeed, high concentrations (36 μmolar) of linoleic or arachidonic acid decreased the apparent affinity two to five-fold. Their effect was much greater for inhibition of binding of 1,25(OH)_2_D than for 25OHD (18). Saturated fatty acids and many other lipid soluble serum substances do not interfere with the binding of DBP to vitamin D metabolites. Physiologic concentrations of unsaturated fatty acids may thus impair the binding of 1,25(OH)_2_D to DBP (18). Whether this transport of fatty acids plays a real role in energy or vitamin D transport is not known.

### DBP and Inflammation

DBP has no direct effects on inflammation. Its ligand, 1,25(OH)_2_D has many immune and inflammatory effects [reviewed in (34, 35)] and DBP inhibits the cellular entry of 1,25(OH)_2_D. DBP, however, is able to bind to membranes and chondroitin sulphated proteoglycans of leucocytes. In association with annexin A2, this binding enhances complement C5a-stimulated chemotactic activity. Quiescent neutrophils do not bind DBP and binding is dependent on prior neutrophil activation. As this activity is linked to activated leucocytes, it may play a role in local inflammation in response to cell damage by whatever mechanism (19, 138, 139). A more extensive discussion on this action of DBP has been published (34, 140) and no further data have recently been published on that topic.

### DBP and Macrophage Activating Factor (MAF)

Several *in vitro* studies suggested that the deglycosylation of DBP can activate DBP to become a macrophage activating factor (MAF). A membrane bound β-galactosidase present on the surface of immune B cells and a sialidase present on T cells would remove two of the three sugar residues of DBP/GC1 protein (141). This DBP would then be able to facilitate the differentiation of monocytes to become osteoclasts and even correct the osteopetrosis phenotype of mice (142). In other circumstances DBP-MAF would activate macrophages in their battle against cancer cells or infections (143, 144). A few clinical trials tested the potential effects of DBP-MAF against HIV infections or cancer. Most of the data on DBP-MAF comes from Yamamoto's laboratory but he had to retract several major papers because of irregularities in the documentation of his data after institutional review [retracted manuscripts included (145, 146)]. Moreover, the availability of nagalase to act as endoglycosylase (and thus to deglycosylate DBP) has been questioned (147). Therefore, there is serious doubt on the existence and role of DBP-MAF despite a large number of publications [reviewed in (34, 148)].

### Polymorphisms of DBP and Disease

The polymorphisms of DBP have been associated with susceptibility or resistance to a large number of chronic conditions, such as osteoporosis (149–151), type 1 and type 2 diabetes (152), thyroid autoimmunity (153), inflammatory bowel disease (154), and chronic obstructive lung disease (155). The exact role of DBP in the pathophysiology of these diseases is however not completely understood [see (156, 157) for an extensive discussion].

In summary, GC/DBP has two major functions:

1. *The transport of all vitamin D metabolites (at a single binding cleft of the A domain of the protein). Due to the combination of high affinity for vitamin D metabolites and a high protein concentration, free concentrations of all vitamin D metabolites are extremely low*.
2. *Tight binding of actin, creating a DPB-Actin complex that avoids actin polymerization in serum after tissue damage*.
3. *Other possible functions of GC/DBP need further validation*.

## Summary and Perspectives

Vitamin D and all its metabolites are bound to a specific vitamin D binding protein, DBP. This discovery was made independently by studying the electrophoretic mobility of antirachitic activity or radiolabeled vitamin D (metabolites) and by studying the polymorphism of a major serum protein called Group-specific Component (GC). We now know that DBP and GC are the same protein and appeared early in the evolution of vertebrates. DBP is genetically the oldest member of the albuminoid family (including albumin, α-fetoprotein, and afamin, all involved in transport of fatty acids or hormones). Some fish use lipoproteins as a sole transport protein but many other fish and (nearly) all amphibia, reptiles, birds, and mammals use DBP. This protein has a single binding site for all vitamin D metabolites and has a high affinity for 25OHD and 1,25(OH)_2_D. This allows creating a large pool of circulating 25OHD, which prevents rapid vitamin D deficiency when the supply of new vitamin D is compromised. DBP also regulates the access of all vitamin D metabolites to cells and tissues. In birds and mammals, DBP combines a high affinity with high concentration, whereas its concentration in early vertebrates is much lower. DBP of higher vertebrates (not amphibians or reptiles) binds with very high affinity actin, creating a 1:1 DBP:actin complex, and thereby preventing the formation of polymeric actin fibrils in the circulation after tissue damage. Megalin is a cargo receptor and is expressed in many epithelial cells, including the luminal border of the renal tubuli. Megalin and cubilin are needed to reabsorb DBP or the DBP-25OHD complex (and many other proteins and their ligands), filtered in the renal glomerulus, thereby preventing the urinary loss of these proteins and 25OHD. Absence of one of these receptors results in rickets, unless the daily supply of vitamin D is able to replace its excessive urinary loss. The total concentrations of 25OHD and 1,25(OH)_2_D in DBP null mice or humans are extremely low (at the limit of detection) but their free concentrations in serum and tissues are probably normal. This does not impair the action of the vitamin D endocrine system on gene expression or tissue action, and therefore is the strongest argument for claiming that the “free hormone hypothesis” also applies to the vitamin D hormone, 1,25(OH)_2_D. The relative importance of free 25OHD compared to total 25OHD is not yet settled. The ongoing standardization of DBP assays and the validation of a direct ELISA for free 25OHD should allow generating the data needed to answer this question in the near future. DBP also transports fatty acids, and unsaturated fatty acids compete with vitamin D metabolites, thereby decreasing the apparent affinity of DBP for 25OHD and especially 1,25(OH)_2_D. DBP can bind to proteoglycans present in the membrane of immune cells and thereby enhance complement C5a-stimulated chemotactic activity of activated neutrophils. This role in inflammation is not fully understood. DBP null mice and a single woman with total absence of DBP are healthy and do not have a clinical phenotype related to vitamin D transport or actin, megalin, or complement binding. DBP is genetically very polymorphic with three frequent alleles (DBP/GC 1f, 1s, and 2) but in total more than 120 different variants. There is a clear North-South gradient of this polymorphism but its health consequences, if any, are not understood. There is a need for standardization of assays for serum DBP as to allow the studies needed to further explore the role of DBP in physiology and diseases. Despite the enormous progress in the deciphering of the structure of DBP and its function, there remains an impressive list of major research questions (Table 4).

**Table 4:** Major remaining research problems and questions.

1	International standard for (gene-specific) DBP and reference method for the measurement of DBP
2	Validation of measurement of free 25OHD and its potential clinical value in comparison with total 25OHD
3	Validation of measurement or calculation of free 1,25(OH)_2_D and its potential physiologic and clinical implications
4	Understanding of the environmental drive (and health implications) for the world-wide gene polymorphism of DBP/GC
5	Full understanding of the implications of actin-DBP binding in health and diseases
6	Understanding of the role of DBP in inflammation
7	Role of megalin and DBP-25OHD uptake, if any, in non-renal cells

## Author Contributions

RB: design and overall writing of the manuscript. FS: genetic origin of DBP and writing of several chapters of manuscript. LA: polymophism of DBP and overall correction of manuscript and references. FR: structure-function analysis of DBP and overall correction of manuscript.

### Conflict of Interest

The authors declare that the research was conducted in the absence of any commercial or financial relationships that could be construed as a potential conflict of interest. The reviewers JS and JSS declared a past co-authorship with one of the authors RB to the handling editor.

## Footnotes

^1^The formula used to calculate free hormone concentration usually refers to Vermeulen et al. (99). In reality, however, the real inventor of the formula is Walter Heyns from the Leuven laboratory of endocrinology (see his formula for calculating free cortisol (100) who taught Vermeulen's assistant (L. Verdonck), during a working visit in Leuven, how to use his formula to calculate free testosterone concentrations. The Vermeulen group later independently validated this calculation by comparing the results with direct measurements of free testosterone (Walter Heyns and Alex Vermeulen, personal communication 2018).
